# Leveraging deep learning for early detection of cervical cancer and dysplasia in China using U-NET++ and RepVGG networks

**DOI:** 10.3389/fonc.2025.1624111

**Published:** 2025-09-12

**Authors:** Baoqing Li, Lulu Chen, Chudi Sun, Jian Wang, Sicong Ma, Hang Xu, Luyao Wang, Taotao Rong, Qun Hu, Jie Wei, Lijuan Lu, Guannan Bai, Zhangdaihong Liu, Peng Luo, Aimin Xu, Li Liu, Guoliu Ye, Lin Zhang

**Affiliations:** ^1^ Department of Laboratory Medicine, The Second Affiliated Hospital of Wenzhou Medical University, Wenzhou, China; ^2^ Department of Gynecology, The First Affiliated Hospital of Wenzhou Medical University, Wenzhou, China; ^3^ School of Computer Science and Technology, Soochow University, Suzhou, China; ^4^ School of Public Health and Preventive Medicine, Monash University, Melbourne, VIC, Australia; ^5^ Suzhou Industrial Park Monash Research Institute of Science and Technology, Suzhou, Jiangsu, China; ^6^ School of Computer Science, The University of Sydney, Sydney, NSW, Australia; ^7^ Department of Gynecology, The First Affiliated Hospital of Bengbu Medical University, Bengbu, Anhui, China; ^8^ Comprehensive Cancer Prevention and Treatment Center, Nantong University Affiliated Jiangyin Hospital, Jiangyin, China; ^9^ Department of Child Health Care, Children’s Hospital, Zhejiang University School of Medicine, National Clinical Research Center for Child Health, Hangzhou, Zhejiang, China; ^10^ Institute of Biomedical Engineering, Department of Engineering Science, University of Oxford, Oxford, United Kingdom; ^11^ Oxford-Suzhou Centre for Advanced Research, Suzhou, Jiangsu, China; ^12^ Department of Oncology, Zhujiang Hospital, Southern Medical University, Guangzhou, China; ^13^ College of Big Data and Software Engineering, Zhejiang Wanli University, Ningbo, China; ^14^ Data Center, Affiliated Hospital of Jiangnan University, Wuxi, China

**Keywords:** cervical cancer screening, public health strategy, deep learning, colposcopy, early diagnosis, resource-limited settings

## Abstract

**Background:**

Cervical cancer is a significant global public health issue, primarily caused by persistent high-risk human papillomavirus (HPV) infections. The disease burden is disproportionately higher in low- and middle-income regions, such as rural China, where limited access to screening and vaccinations leads to increased incidence and mortality rates. Cervical cancer is preventable and treatable when detected early; this study utilizes deep learning to enhance early detection by improving the diagnostic accuracy of colposcopic image analysis.

**Objective:**

The aim of this study is to leverage deep learning techniques to improve the early detection of cervical cancer through the enhancement of colposcopic image diagnostic accuracy.

**Methods:**

The study sourced a comprehensive dataset of colposcopic images from The First Affiliated Hospital of Bengbu Medical University, with each image manually annotated by expert clinicians. The U-NET++ architecture was employed for precise image segmentation, converting colposcopic images into binary representations for detailed analysis. The RepVGG framework was then applied for classification, focusing on detecting cervical cancer, HPV infections, and cervical intraepithelial neoplasia (CIN). From a dataset of 848 subjects, 424 high-quality images were selected for training, with the remaining 424 used for validation.

**Results:**

The deep learning model effectively identified the disease severity in colposcopic images, achieving a predictive accuracy of 83.01%. Among the 424 validation subjects, cervical pathology was correctly identified in 352, demonstrating high diagnostic precision. The model excelled in detecting early-stage lesions, including CIN I and CIN II, which are crucial for initiating timely interventions. This capability positions the model as a valuable tool for reducing cervical cancer incidence and improving patient outcomes.

**Conclusion:**

The integration of deep learning into colposcopic image analysis marks a significant advancement in early cervical cancer detection. The study suggests that AI-driven diagnostic tools can significantly improve screening accuracy. Reducing reliance on human interpretation minimizes variability and enhances efficiency. In rural and underserved areas, the deployment of AI-based solutions could be transformative, potentially reducing cervical cancer incidence and mortality. With further refinement, these models could be adapted for broader population screening, aiding global efforts to eliminate cervical cancer as a public health threat.

## Introduction

Cervical cancer remains a major global health challenge, with over half a million new cases diagnosed annually and significant mortality, especially in low- and middle-income countries (1). The main methods of cervical cancer screening include human papillomavirus test (HPV test) and liquid based cytology test (LCT). LCT image can accurately and intuitively show the morphological characteristics of cervical cells and is also an accurate method to judge the precancerous lesions of cervical cancer (2). Despite the availability of various preventive measures and screening techniques, such as human papillomavirus (HPV) vaccinations and routine Pap smear tests (3, 4), manual screening processes are not always highly accurate (5), potentially leading to delayed diagnoses of related pathological changes (6). Colposcopy is an important diagnostic tool in women’s health care and plays an important role, especially in the early detection and prevention of cervical cancer. A low-power microscope that uses acetic acid and Lugol solution to carefully examine the cervix, vagina, and vulva and provide a zoomed view of these areas, enabling healthcare professionals to identify and assess abnormalities that may not be visible to the naked eye. In a large trial of low-grade abnormalities, the initial colposcopy was only 53% sensitive to detecting high-grade disease within the following 2 years. Studies have shown low agreement between colposcopic impressions of disease and final histology. The use of multiple biopsies can improve the accuracy of colposcopy diagnosis. Although colposcopy is practiced by many clinicians (including senior practitioners, gynecologists, gynecologic oncologists, etc.), the standardization of the procedure, the necessary training, and the ongoing development and maintenance of colposcopy skills are generally poor. There is also ample evidence that colposcopy has significant executive-to-practitioner variability and poor reliability. In addition, a lot of complicated colposcopy and image reading increase the workload of clinicians. Furthermore, in the context of uneven development, many countries and regions face challenges in training or retaining specialized colposcopists and cervical cytopathologists (7). Consequently, the development of a more accurate and cost-effective method for cervical cancer screening has emerged as a primary challenge for the early detection of the disease.

Artificial intelligence (AI), particularly deep learning (DL), has emerged as a powerful tool to address these challenges. Deep learning models excel at analyzing large datasets of medical imagery and offer high precision in detecting pre-cancerous conditions, potentially reducing the need for human expertise and improving diagnostic consistency (2, 8–10). Recent studies have demonstrated the effectiveness of AI models, such as transfer learning for cervical cell image classification and U-net-based models for cancer screening using medical images (11–16). These advancements hold promise for reducing the diagnostic burden in both high- and low-resource settings.

In the diagnosis and treatment of gynecological malignant tumors, the imaging information of the lesion site can provide an important diagnostic basis for clinicians. With the popularization and continuous development of imaging technology, a large number of medical images need to be managed reasonably and efficiently. The novelty of this study lies in its focus on colposcopic image analysis using deep learning, rather than relying solely on traditional cytological or histological methods (7). While AI has been applied to cervical cancer diagnosis before, most efforts have cantered on cytological screening. Our work, by contrast, leverages deep learning algorithms to automatically interpret colposcopic images—a more direct, non-invasive screening tool that can be particularly effective in regions with limited access to trained cytologists or pathologists. This approach introduces an innovative pathway for early detection, offering a viable solution that can be more easily integrated into healthcare systems with constrained resources.

Furthermore, our contributions include the development and validation of a deep learning model that combines advanced image segmentation techniques and transfer learning strategies. These enhancements allow for more accurate classification of cervical abnormalities and can significantly reduce human error in the screening process. The model was validated on multiple independent datasets, ensuring robustness and generalizability across diverse clinical environments. By focusing on improving diagnostic accuracy in low-resource settings, our system offers a practical, cost-effective solution that addresses the global disparity in cervical cancer outcomes.

This research aligns with global health initiatives aimed at reducing cancer-related mortality by expanding access to cutting-edge diagnostic tools, especially in underserved populations. The use of AI in colposcopy represents a promising step forward in the effort to democratize healthcare and deliver more equitable outcomes worldwide.

## Methods

### Data preparation

**Figure 1:**
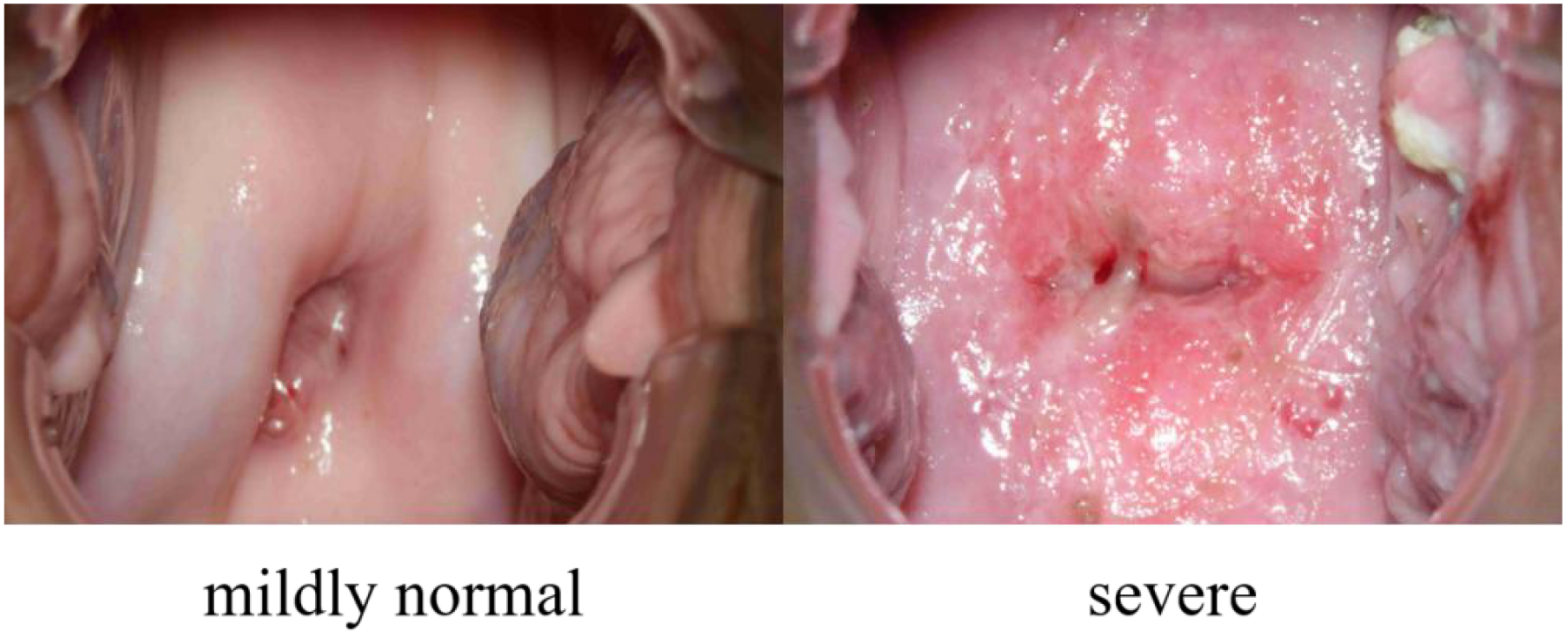
Sample images.

Our dataset, collected from the Department of Gynecology, the First Affiliated Hospital of Bengbu Medical University, Anhui, PR China, under ethical approval (Approval Number: the First Affiliated Hospital of Bengbu Medical University 2024【427】), consists of 7,612 colposcopic images from 848 distinct female subjects, with each subject contributing multiple images. These images represent 27 different pathological conditions, and it is common for a single colposcopic image to be associated with multiple diseases. To streamline classification, these conditions have been grouped into two primary categories: “mildly normal” and “severe”. The “mildly normal” category includes conditions that are asymptomatic or do not require further medical attention, while the “severe” category encompasses conditions that necessitate additional treatment. Accurately distinguishing between these two categories can play a crucial role in early screening, helping patients promptly identify the need for further medical care, thereby improving overall health management. An example of this is shown in **Figure 1**.

**Figure 2:**
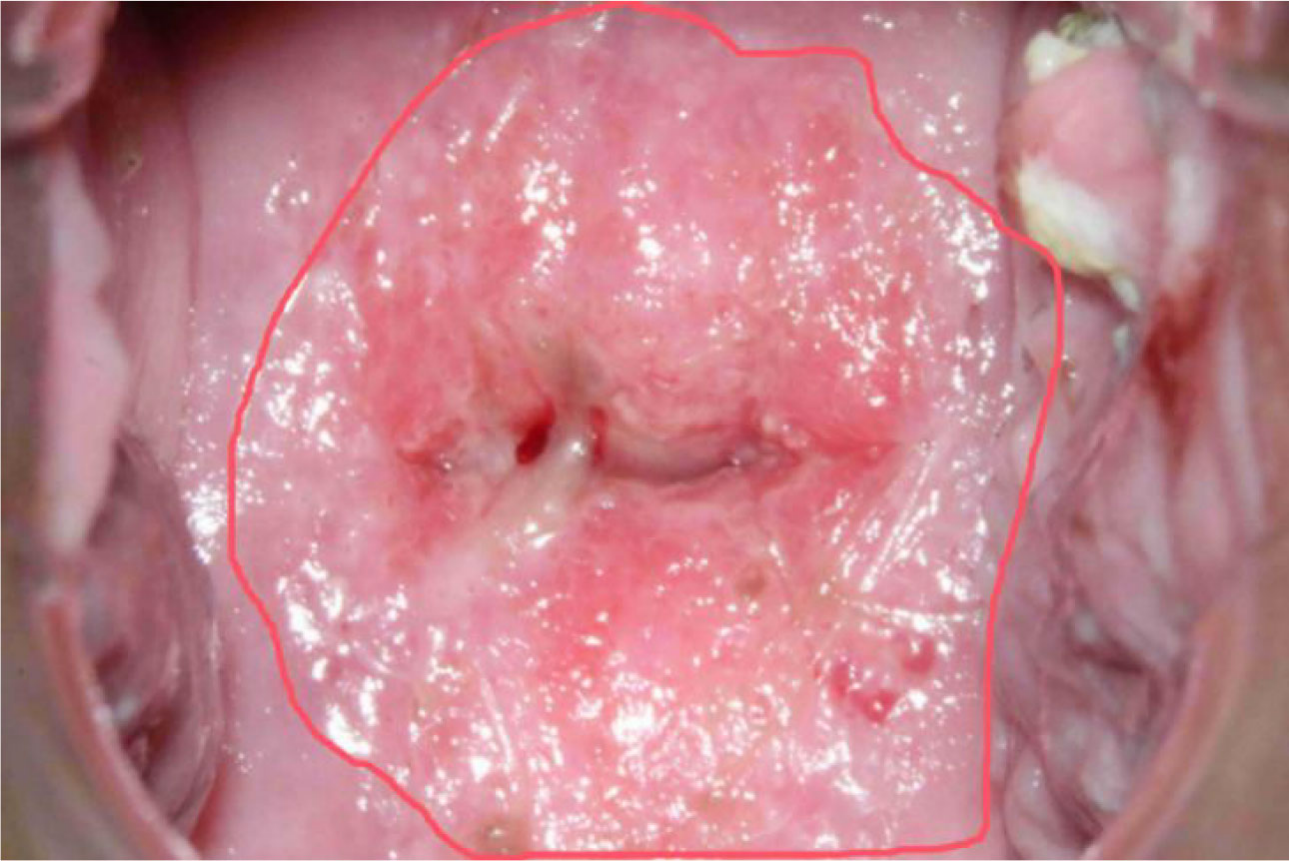
Example of drawing segmentation regions in the image.

In colposcopic image analysis, it’s common to find that images may contain limited feature information from the regions of interest, leading to potential bias toward non-critical areas during the model training process. This can result in the model learning less relevant information. To address this, we employed a sampling methodology that leverages closed curves to segment areas of interest based on their morphological characteristics. As depicted in **Figure 2**, red closed curves are used to highlight potential disease regions.

A total of 51 colposcopic images underwent rigorous screening and pixel-level annotation by two experienced colposcopists. While formal inter-rater statistics were not calculated, annotations were completed in consensus based on clinical diagnostic criteria. These annotated images were used solely for segmentation model training.

Of these, 235 images were classified as indicative of severe conditions, and 5 images were determined to be clearly normal. Additionally, through an image screening process that aligns each image with its respective disease type, 184 colposcopic images were identified as having features that, while not overtly abnormal, were confirmed as normal after examination. This brings the total number of images classified as normal and non-diseased to 189, resulting in a class ratio of approximately 1:1.24 (severe:normal). This stratified sampling technique helps to address data imbalance, improving model training in subsequent phases.

Note: The 51 annotated images refer to a segmentation subset used for U-Net++ training. In contrast, the classification dataset (424 training + 424 validation images) was constructed separately through stratified sampling of diagnostically validated images from the full set of 7,612.

### Process and methods

**Figure 3:**
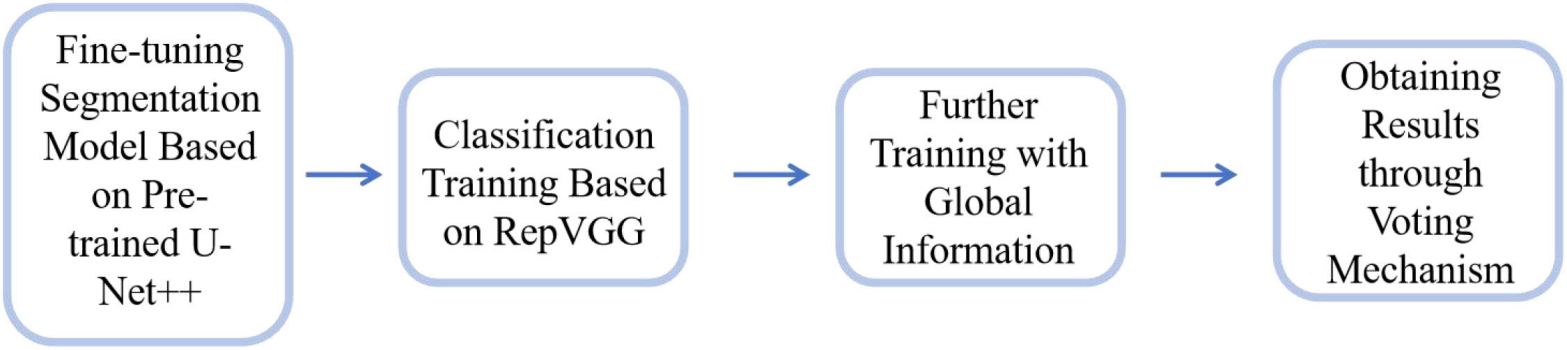
Model framework workflow.

This study establishes a model framework for early cervical cancer screening using convolutional neural networks (CNNs). The operational workflow of the model is illustrated in **Figure 3** and comprises the following stages:

1. Pre-training and Segmentation: To address the limited availability of segmentation annotation data in our dataset, we utilized the publicly available AnnoCerv dataset (17) for pre-training on a U-Net++ model (18). This pre-trained network was subsequently fine-tuned with our dataset, enabling the model to achieve faster convergence.
2. Feature-based Classification: For the regions of interest identified by the segmentation model, we employed the RepVGG model (19) to perform classification training. This allowed the model to focus on disease-relevant features.
3. Incorporating Global Information: Since the classification was initially based solely on feature regions, the model could develop biases. To mitigate this, we incorporated global context by including the original, unsegmented images along with the segmented feature-only images in the training process. This ensured that the model retained both local and global information.
4. Final Model Output: The final model uses a voting mechanism that considers predictions from both the original and segmented images across multiple iterations. The category with the highest voting weight is selected as the final model output, ensuring robust and reliable classification results.

### Methodology

Initially, we employed the U-Net++ architecture to perform segmentation on our dataset. The segmentation quality was quantified using the Intersection over Union (IoU), defined as: $IoU=\frac{\left|A\cap B\right|}{\left|A\cup B\right|}$ , where $\left|A\cap B\right|$ represents the number of pixels in the intersection of the predicted segmentation result A and the ground truth B, and $\left|A\cup B\right|$ denotes the number of pixels in the union of A and B.

**Figure 4:**
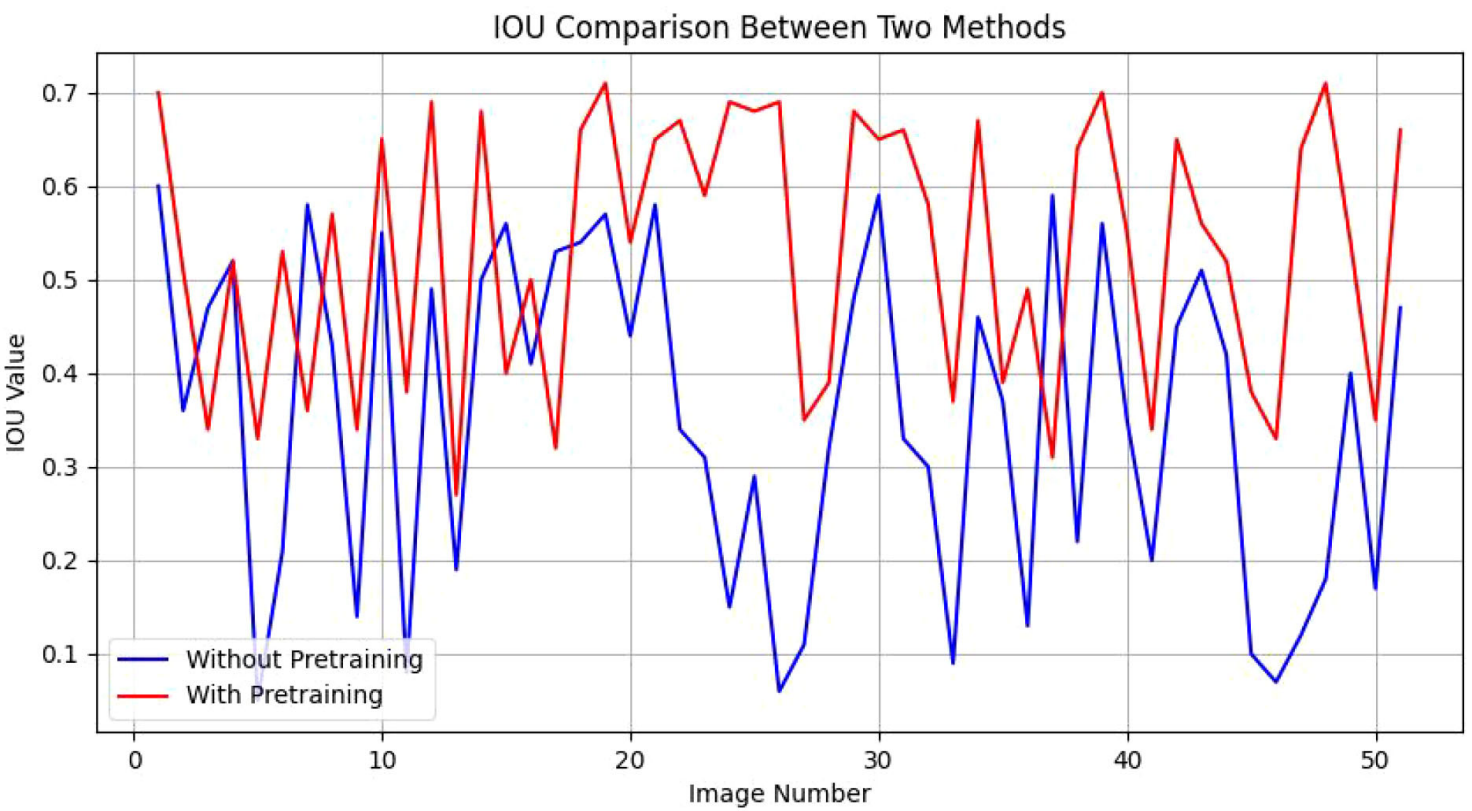
IOU comparison between two methods.

When trained directly on our entire dataset, the network’s performance yielded an IoU range of 0.05 to 0.60 across 51 segmented images, reflecting suboptimal segmentation quality. To address this, we utilized the publicly available AnnoCerv dataset for pre-training, followed by fine-tuning on our specific dataset. This enhanced the model’s performance, with an improved IoU range of 0.27 to 0.71 for the same 51 segmented images, a significant improvement over the direct training approach. As shown in **Figure 4**, we compare the IOU values of each picture. Our observations more intuitively reveal a phenomenon: in the majority of images, the segmentation performance of models with pre-training is only slightly better than that of models without pre-training. However, in more than ten images, the segmentation performance of the pre-trained models significantly surpasses that of the non-pre-trained methods. This observation, to a certain extent, validates the effectiveness of the strategies we have employed.

The segmented images primarily captured regions associated with potential pathological features. These segmented images were then employed for classification training using the RepVGG network. The classification training on our dataset, consisting of 424 images, achieved an accuracy of 84.19%. In the remaining set of validation images, there are 419 instances classified as ‘severe’. In contrast, only a mere five images are categorized as ‘mildly normal.’ The likely reason for this disparity within the dataset is that the majority of individuals undergoing colposcopic examinations are presumed to be symptomatic, suggesting a potential diagnosis. Consequently, the scarcity of ‘mildly normal’ images may be attributed to this bias toward symptomatic patients. When applied to disease assessment of 424 women in validation subset, the model’s accuracy was 74.05%, which fell short of the threshold required for clinical applicability. This suggests that models trained solely on regions of interest may miss critical global information in unseen test data.

To address this limitation, we integrated the original images with their corresponding segmented regions for joint training, improving the model’s training accuracy to 88.44%. This approach also enhanced the disease classification accuracy for the 424 women in validation subset to 81.83%.

Additionally, we introduced a voting mechanism to further refine the model’s output. For each patient, an original image (Image 1) is passed through the segmented model to isolate the region of interest (Image 2). Both Image 1 and Image 2 are then flipped horizontally, producing Images 3 and 4, respectively. To account for varying real-world lighting conditions, the luminance of these images is adjusted, resulting in Images 5 and 6. The voting process, based on the weights assigned to each image (as detailed in **Table 1**), determines the final classification. An image is classified as belonging to a particular category if the voting score for that category exceeds 0.5. For patients with multiple images, each original image is assigned equal weight, and the final disease status is determined by the collective results of all images.

**Table 1:** Corresponding weight information of images.

**Figure**	1	2	3	4	5	6
**Weight**	0.2	0.25	0.15	0.2	0.11	0.09

This voting mechanism increased the overall accuracy in validating the disease status of all women to 83.01%, demonstrating a significant improvement in the model’s diagnostic performance.

The voting weights assigned to each image variant (e.g., flipped, brightness-adjusted) were determined empirically through iterative tuning. While not obtained via formal grid search, these weights reflect their relative contributions to improved ensemble classification accuracy on the validation set.

## Results

To demonstrate the overall efficacy of our algorithmic framework, we conducted comparative experiments with several state-of-the-art methods and performed ablation studies on our approach. Since our ultimate goal is to achieve a classification result, the neural network methods we compared include the conventional models such as VGG-16, ResNet-50, and Densenet-121. All models were trained with consistent hyperparameters using PyTorch 2.1.0 and torchvision v0.15.0 on an NVIDIA RTX 4070 Ti SUPER GPU(16 GB VRAM), with 80 training epochs (no early stopping), batch size 8, Adam optimizer (learning rate = 0.001), and categorical cross-entropy loss.

Our ablation experiments investigated several configurations: models that do not employ pre-trained segmentation procedures, models that entirely omit the segmentation process, and models that utilize segmentation but forgo the voting mechanism. Notably, the model excluding both the segmentation process, and the voting mechanism corresponds to the original RepVGG model. The results of these experiments are summarized in **Table 2** below.

**Table 2:** Comparative experiments.

Methods	Accuracy (%)
VGG	72.40
Res-net	75.94
Densenet	72.87
Without pre-trained (WOPT)	74.05
Without segmentation (WOS)	75.70
Without Voting Mechanism (WOVM)	81.83
Repvgg	74.76
Ours	83.01

**Figure 5:**
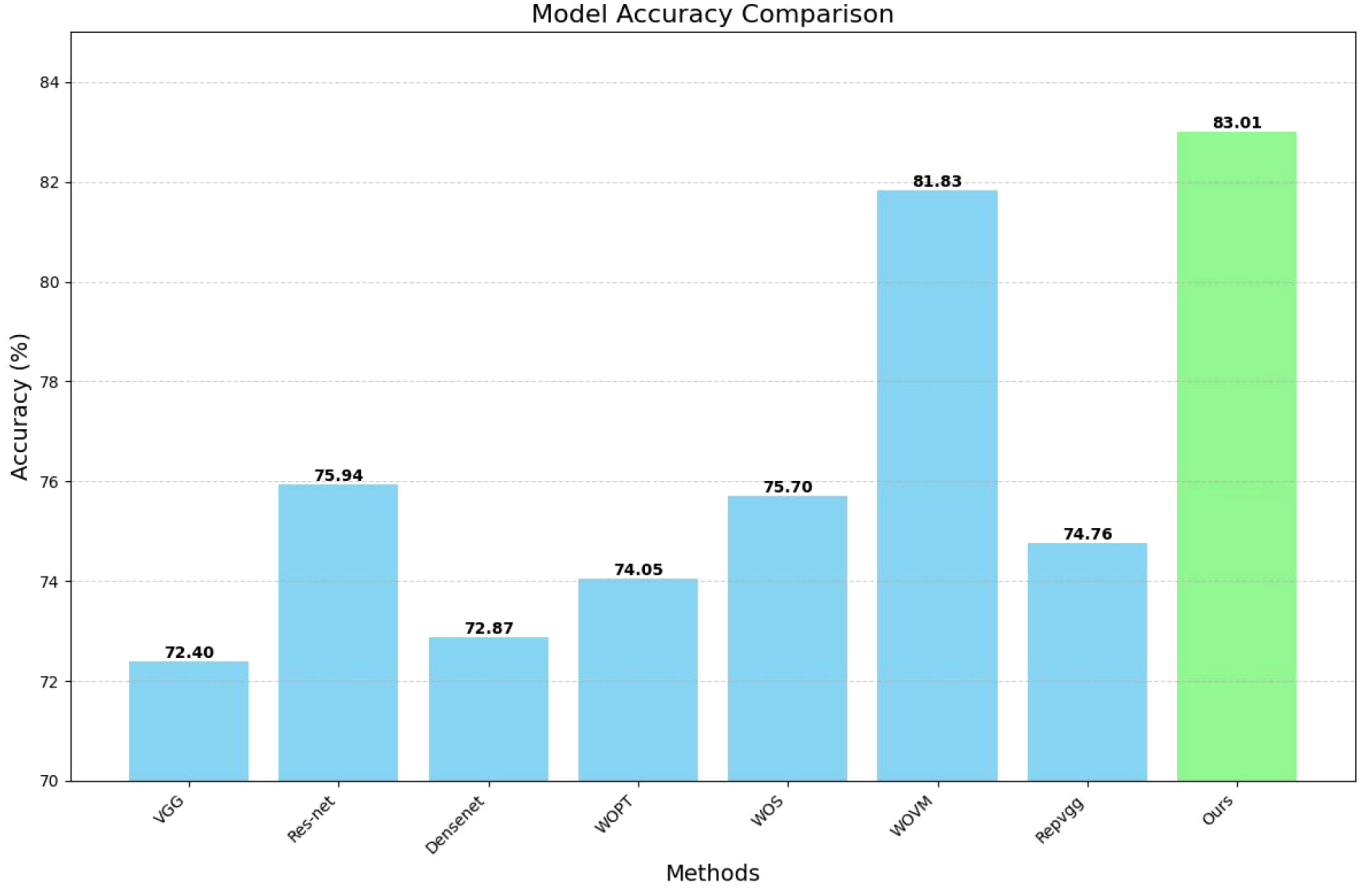
Comparative experiments of methods.

To more clearly present our data, we have plotted a bar chart as shown in **Figure 5** below:

An analysis of the comparative results reveals that the impact of various classical classification network models on the final image classification outcome is generally minimal. However, this effect becomes more pronounced when using a limited amount of segmented image data, particularly if no additional data is used for pre-training. Interestingly, the model that excludes additional data for segmentation sometimes yields results that are slightly inferior to those of the original classification network. In contrast, the extraction of key information areas through the segmentation operation prior to classification significantly enhances the model’s performance. Consequently, our experimental design methodology demonstrates a substantial degree of effectiveness and robustness.

**Figure 6:**
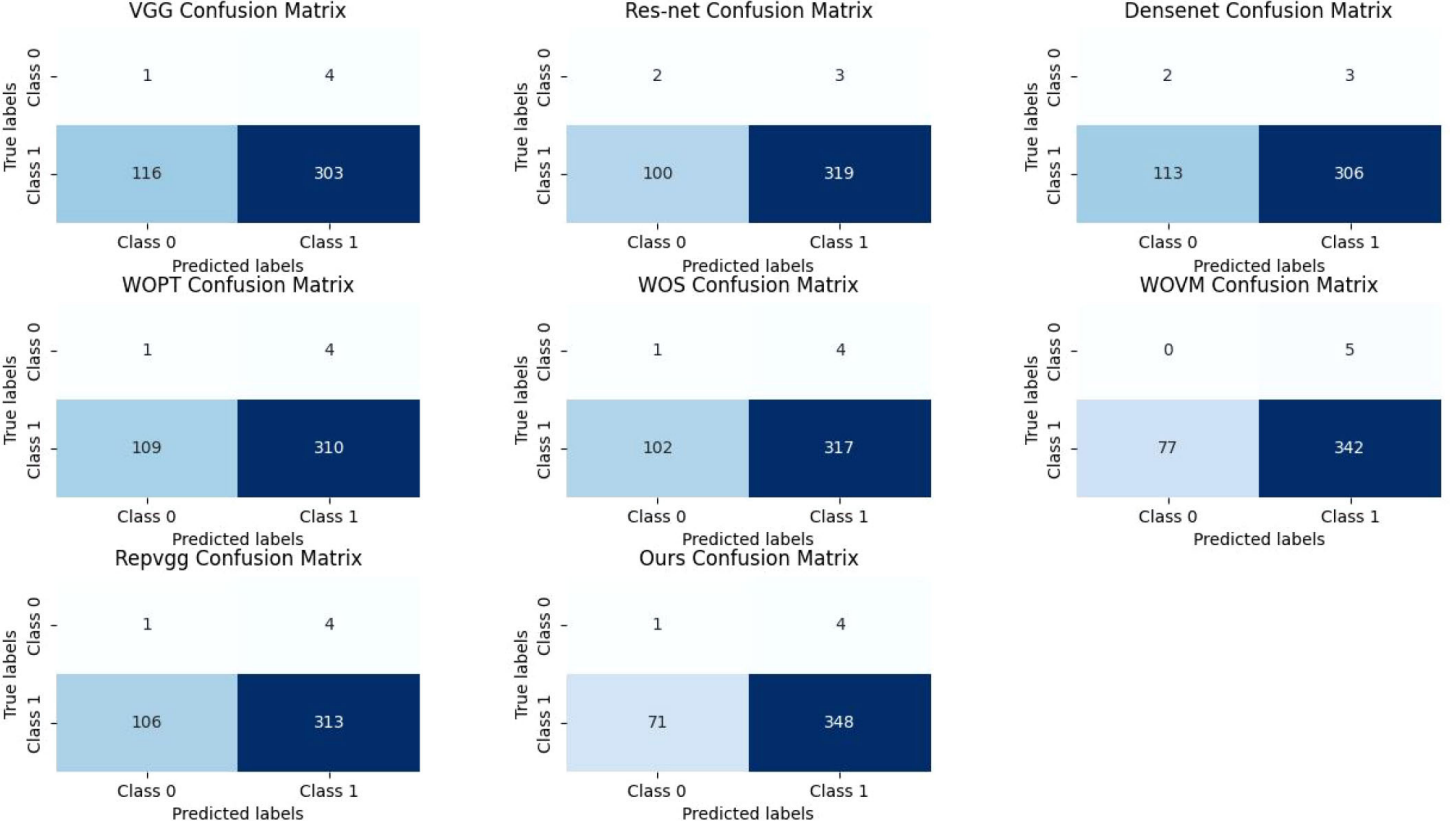
Confusion matrix.

In fact, in the classification of images, the most intuitive way to reflect the effectiveness of the model is to verify the accuracy of the set, and at the same time, we draw its confusion matrix, as shown in **Figure 6**. Although the number of images classified as “mildly normal” is relatively small, making it difficult to accurately assess the model’s recognition rate for this category from the experiments, we can observe from the figures that our model’s misclassification of “severe” images is significantly lower than that of other models. In the context of medical research, the tolerance for misclassifying disease-free images as diseased is considerably higher than that for misclassifying diseased images as disease-free, as the consequences of overlooking a disease can be far more severe than the inconvenience caused by false positives. This is particularly crucial in diagnostic settings where early and accurate detection of diseases can significantly impact patient outcomes. From this standpoint, our model’s ability to minimize the misclassification of diseased images as disease-free demonstrates its vastly superior performance compared to other models, which is a critical advantage in clinical applications.

## Discussion

Cervical cancer remains the leading cause of cancer-related death among women in developing countries. However, the disease is highly curable when detected early through effective screening. In this study, we developed a novel, automated cervical cancer screening system based on deep learning, aiming to assist clinicians in identifying early signs of precancerous lesions and cancer via colposcopy image analysis. By integrating advanced image processing and machine learning techniques, the system demonstrates the potential to improve diagnostic accuracy, reduce clinician workload, and minimize misdiagnosis, particularly in resource-limited settings where trained specialists may be scarce.

The proposed system incorporates segmentation-guided classification, enabling it to extract and analyze key regions of interest in colposcopic images. Our experimental results show that incorporating global information, using pre-trained models for segmentation, and applying a voting mechanism significantly enhance the model’s performance. Specifically, the final model achieved an overall accuracy of 83.01% in a test cohort of 848 women, supporting the robustness and effectiveness of the design. Moreover, we found that accurate localization and segmentation of diagnostic regions had a direct and substantial impact on downstream classification performance, emphasizing the necessity of precise image preprocessing in automated screening workflows.

Despite the encouraging results, we acknowledge several limitations that warrant further investigation. First, although the study involved multi-institutional collaboration, the dataset was collected from a single medical center. This introduces potential bias related to device-specific settings and clinical protocols, which may affect the model’s generalizability. To address this, we are actively working with additional clinical sites to build a multi-center dataset for comprehensive external validation.

Second, the validation cohort had a limited number of “mildly normal” cases, reflecting the symptomatic referral nature of the data source. We recognize that this may influence specificity and are currently enriching the dataset to better reflect the full spectrum of screening populations.

Third, while the segmentation module plays a critical role in model performance, only 51 images were pixel-wise annotated for quantitative evaluation. Although this subset enabled proof-of-concept validation, we plan to expand the number of annotated samples to allow more statistically robust assessments.

Additionally, the voting weights used in the ensemble process were selected empirically. Future work will explore formal optimization strategies, such as grid search or Bayesian optimization, to determine optimal fusion parameters.

Regarding evaluation metrics, although we primarily report classification accuracy—a widely accepted benchmark in deep learning classification tasks—we acknowledge that additional diagnostic metrics such as ROC curves, AUC, sensitivity, specificity, and precision-recall curves provide complementary insights. These will be reported in follow-up studies to provide a more comprehensive assessment of diagnostic performance.

Furthermore, although we explored visual interpretability tools (e.g., Grad-CAM) internally to assess model decision-making transparency, these results were excluded due to space limitations and will be detailed in subsequent publications.

Finally, while recent AI-based cervical screening tools provide important contributions to the field, they differ in screening modality (e.g., cytology, HPV biomarkers) and diagnostic focus. Our work, which centers on colposcopic image analysis, offers a complementary approach that can be integrated into broader screening pathways.

In summary, this study presents a practical, scalable, and interpretable AI-assisted cervical cancer screening system that demonstrates strong diagnostic performance and promising real-world applicability. With continued refinement—particularly in terms of data diversity, interpretability, and evaluation metrics—this framework may help expand access to high-quality screening, especially in under-resourced regions, and ultimately contribute to the global effort to reduce cervical cancer incidence and mortality.

## Conclusion

This study presents a novel and effective automated screening system for cervical cancer based on deep learning, achieving an accuracy of 83.01% in testing. By leveraging advanced image processing and machine learning techniques, the system enhances screening specificity and accuracy, reduces clinician workload, and mitigates subjective biases. It holds promise for resource-limited settings, significantly improving early diagnosis and reducing cervical cancer incidence. Despite its success, challenges such as handling blurry images and ensuring system robustness remain. Future work should focus on larger datasets and more advanced model architectures to further enhance performance and accessibility globally.

## Data Availability

The original contributions presented in the study are included in the article/supplementary material. Further inquiries can be directed to the corresponding authors.
